# Integration of lncRNAs, Protein-Coding Genes and Pathology Images for Detecting Metastatic Melanoma

**DOI:** 10.3390/genes13101916

**Published:** 2022-10-21

**Authors:** Shuai Liu, Yusi Fan, Kewei Li, Haotian Zhang, Xi Wang, Ruofei Ju, Lan Huang, Meiyu Duan, Fengfeng Zhou

**Affiliations:** 1 College of Computer Science and Technology, and Key Laboratory of Symbolic Computation and Knowledge Engineering of Ministry of Education, Jilin University, Changchun 130012, China; 2 College of Software, and Key Laboratory of Symbolic Computation and Knowledge Engineering of Ministry of Education, Jilin University, Changchun 130012, China

**Keywords:** bioinformatics, metastatic melanoma, multimodal information fusion, lncRNA, feature selection

## Abstract

Melanoma is a lethal skin disease that develops from moles. This study aimed to integrate multimodal data to predict metastatic melanoma, which is highly aggressive and difficult to treat. The proposed EnsembleSKCM method evaluated the prediction performances of long noncoding RNAs (lncRNAs), protein-coding messenger genes (mRNAs) and pathology images (images) for metastatic melanoma. Feature selection was used to screen for metastatic biomarkers in the lncRNA and mRNA datasets. The integrated EnsembleSKCM model was built based on the weighted results of the lncRNA-, mRNA- and image-based models. EnsembleSKCM achieved 0.9444 in the prediction accuracy of metastatic melanoma and outperformed the single-modal prediction models based on the lncRNA, mRNA and image data. The experimental data suggest the importance of integrating the complementary information from the three data modalities. WGCNA was used to analyze the relationship of molecular-level features and image features, and the results show connections between them. Another cohort was used to validate our prediction.

## 1. Introduction

Melanoma is a type of malignant skin cancer, and its incidence rate has increased rapidly in recent decades [1,2,3]. The melanoma incidence rate in Canada is 122.9 cases per million person a year [4]. The melanoma mortality rate in the U.S. increased by 7.5% from 1986 to 2013 [5]. Early detection and appropriate treatment of non-metastatic melanoma may decrease the mortality rate and substantially increase survival [6].

Computational models are widely used in the field of disease diagnosis and prognosis. Systems biology models can incorporate various data sources such as mechanistic details of biological mechanisms, inter-patient variability and drug–target interactions into the translational research [7]. Verma et al. fine-tuned the model to predict the liver regeneration process by integrating signaling mechanisms and cellular functional state transitions [8]. Compared with other studies, they performed the liver failure classification to characterize the response of recovery and failure. Milberg et al. developed a quantitative systems pharmacology (QSP) model for the combination immunotherapy specific to melanoma [9]. With the development of high-throughput sequencing and biological technologies, a huge amount of biomedical data is being rapidly accumulated and machine learning approaches are also actively utilized.

The detection of metastatic melanoma using machine learning algorithms is a clinically useful and computationally challenging task. Several machine learning methods delivered promising prediction performances for metastatic melanoma. Bellomo et al. employed classifier logistic regression to combine clinicopathologic and gene expression data for the detection of metastatic melanoma in sentinel lymph nodes [10]. Garg et al. trained a random forest model with the screened signature genes to detect metastatic melanoma [11]. Mancuso et al. predicted metastasis in melanoma patients with high and low risk of metastasis by serum cytokines and Breslow thickness [12]. Shepelin et al. used the SVM algorithm to identify 44 characteristic signaling pathways associated with melanoma metastasis [13].

Melanoma may be detected by machine learning methods. Melanoma develops from moles, and its early detection through the ABCDE criteria may generate some false negatives depending on the experience of the practicing dermatologists [14]. There are two main categories of machine learning-based melanoma detection methods, i.e., image-based and OMIC-based methods [15,16,17,18]. Adytia et al. proposed a novel transfer learning method to classify skin lesions based on the internet of health things [19]. In addition to the classification task, lesion segmentation is another important machine learning task for detecting melanoma [20,21,22]. Rasmiranjan et al. optimized a set of hyperparameters of a fully convolutional encoder–decoder network (FCEDN) to segment skin cancer lesions [23]. Tang et al. employed end-to-end multistage UNets to segment skin lesions accurately [24]. Afsah et al. explored the feasibility of using hybrid textural analysis to segment and classify skin cancers based on dermoscopic images [25].

OMIC data provide a molecular-level view of melanoma, and the detected biomarkers facilitate the understanding of the onset and progression mechanisms of melanoma. Lai et al. selected the fully connected melanoma subnetwork with the best modularity score and proposed an autoencoder-based deep learning network to detect different melanoma subgroups using the genomic data in The Cancer Genome Atlas [26]. Wei revealed 798 differentially expressed genes of melanoma and built a support vector machine (SVM)-based classifier using the top 110 biomarker genes to achieve at least 0.944 in accuracy across three independent datasets [27]. Aigli et al. proposed an ensemble dimensionality reduction technique to estimate melanoma patient prognosis in a large cohort [28].

Long noncoding RNAs (lncRNAs) may act as competing endogenous RNAs (ceRNAs) and are widely involved in tumor onset and progression [29]. Multiple previous investigations have demonstrated that lncRNAs have a close relationship with the prognosis of melanoma [30,31,32]. Yan et al. screened 61 lncRNAs associated with melanoma prognosis and built a weighted risk score model based on seven key candidate lncRNAs [33]. The lncRNA U731166 was also observed to be upregulated during the migration and invasion of melanoma and even played a role in developing vemurafenib resistance [34].

Both image and OMIC data provide complementary information about melanoma, and the integrated analysis of multimodal data is important to fully utilize these different data modalities [35,36]. Both image and OMIC data have large numbers of features, and most do not contribute to detecting melanoma. Such “large p small n” datasets may lead to prediction model overfitting [37]. Feature selection is one of the methods used to tackle this challenge [38,39,40].

This study proposes an ensemble detection algorithm, EnsembleSKCM, for metastatic melanoma by integrating the data sources of lncRNAs, protein-coding mRNAs and pathology images. The extremely high dimensionalities of lncRNAs and mRNAs were screened for redundancies by feature selection algorithms. The image-extracted cell features were combined with the results of lncRNAs and mRNAs for the final classification between metastatic and non-metastatic melanoma samples. The experimental data support the necessity of integrating multimodal data for the detection of metastatic melanoma. The Python source code and the multi-modal datasets are freely available at http://www.healthinformaticslab.org/supp/, accessed on 4 September 2022.

## 2. Materials and Methods

### 2.1. Summary of Datasets

This study retrieved the features of pathology images, lncRNAs and mRNAs of melanoma from The Cancer Genome Atlas (TCGA) database [41,42]. The mRNA expression refers to the expression level of the corresponding gene. FPKM was used to normalize the transcript expression data. The dataset’s metadata are shown in Supplementary Table S1. This TCGA–SKCM cohort selected patients with a diagnosis of primary metastatic cutaneous melanoma or metastatic melanoma of an unknown primary, and they were also required to have had no previous systemic therapy (except that adjuvant interferon-α ≥ 90 days prior was permitted) [43]. This study investigated the integrated analysis of metastatic melanoma based on multi-modality data sources, i.e., lncRNA, mRNA and pathology images. Therefore, the samples without regional lymph node metastasis information were removed. There were 414 melanoma samples with regional lymph node metastasis information, lncRNAs, mRNAs and images in the TCGA database. The number of primary melanoma samples was 235 and that of metastasis melanoma samples was 179. Multiple samples may be extracted from one patient and one of these samples from the same patient was randomly chosen for further analysis. There were 411 remaining patients, among whom there were 257 males and 154 females. The majority (392) of this cohort was white people, and there were only 12 Asian and 1 black or African American. This cohort consisted of only 19 patients under the age of 30. The remaining patients included 191 and 183 patients under and over 60-years-old, respectively. Some samples did not have information on sex, ethics or age.

**Figure 1:**
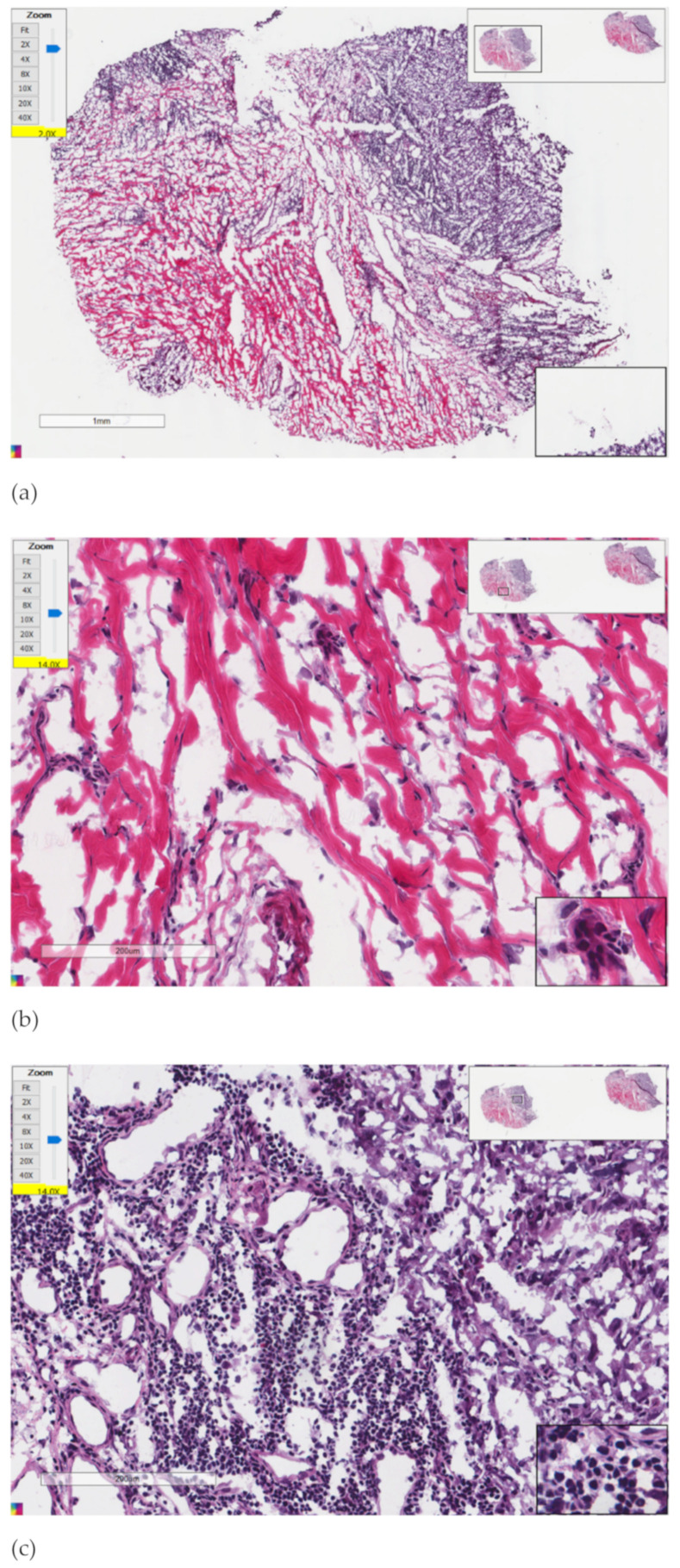
The example pathology image and its randomly cropped patches. (**a**) Shows the whole pathology image; (**b**,**c**) are patch images cropped randomly from (**a**) to show the detailed information.

A binary classification between primary melanoma (*n* = 0, positive samples) and melanoma with regional lymph node metastasis (*n* > 0, negative samples) was investigated. A sample was a melanoma patient, and a feature was an lncRNA’s expression level, an mRNA’s expression level or a cell type’s percentage within the pathological image in this study. Each sample had 6919 lncRNA features and 19051 mRNA features. A feature in a sample was the expression level of the corresponding lncRNA or mRNA gene, or the percentage of a cell type in the pathological image of that sample. The annotations were generated by UCSC for the Dec. 2013 (GRCh38/hg38) assembly of the human genome. For the processing of missing values, all of the 411 samples were considered (including both primary and lymph node metastatic melanoma samples). If the ratio of missing values of a feature was greater than 50%, this feature was removed. The remaining missing values were filled with 0, assuming that the sequencing technology cannot detect the low expression levels of these genes. We did not process the missing values in the primary and metastatic melanoma samples separately, to avoid the case that these two groups of samples may have different features. Eight features were retrieved from the pathology images to describe the percentages of lymphocyte infiltration, monocyte infiltration, necrosis, neutrophil infiltration, normal cells, stromal cells, tumor cells and tumor nuclei. The detection and counting of the different cell types were conducted using the CellProfiler software and the percentages of these cell types were calculated as the representative features for the pathology images [44]. After the preprocessing step, there were 1716 lncRNAs, 1827 mRNAs and 8 image features. The example pathology images are shown in Figure 1 by the freeware ImageScope version 12.4.6 [45]. The image came from the sample “TCGA-BF-A1Q0–01A-02-TSB”. The scale bar of Figure 1a is 1 mm and the scale bars of Figure 1b,c are 200 μm. Figure 1b,c were cropped randomly from (a). This dataset was denoted as “TCGA-SKCM”. 

We screened the GEO database and found only one transcriptomic cohort (GSE59455) of metastatic melanoma for further validation of the experimental results in the above sections. We did not find a cohort with both pathological images and RNA-seq transcriptomes. The GSE59455 dataset was an array-based transcriptomic profile and consisted of 141 samples. It does not have as detailed metadata as the TCGA–SKCM dataset. The samples without information on primary or metastatic cancers were removed, and the remaining samples consisted of 17 primary cancers and 43 metastatic cancers. The array-based GSE59455 dataset and the RNA-seq-based transcriptomic dataset from the TCGA database had large differences in both the feature list and the expression patterns. Only 2 lncRNAs and 198 mRNAs overlapped between the GSE59455 and the TCGA melanoma datasets, while the optimal EnsembleSKCM model used 200 lncRNAs and 200 mRNAs from the RNA-seq-based transcriptomic profiles.

### 2.2. Performance Measurements

The classification model was evaluated by five widely used measurements, i.e., accuracy, F1-score, precision, recall and AUC. Assume that P and N are the numbers of positive and negative samples, respectively. The numbers of correctly predicted positive and negative samples are true positive (TP) and true negative (TN). False positive (FP) and false negative (FN) are the numbers of incorrectly predicted positive and negative samples. The performance measurements were defined as follows.

(1) $$Accuracy=\frac{TP+TN}{TP+FN+TN+FP}$$

(2) $$Precision=\frac{TP}{TP+FP}$$

(3) $$Recall=\frac{TP}{TP+FN}$$

(4) $$F1=\frac{2*Precision*Recall}{Precision+Recall}$$

AUC is the area under the ROC curve, which is a good parameter-independent measurement for a binary classification model. A stratified 5-fold cross-validation (S5FCV) strategy was used to evaluate the models.

### 2.3. The Proposed EnsembleSKCM Method 

**Figure 2:**
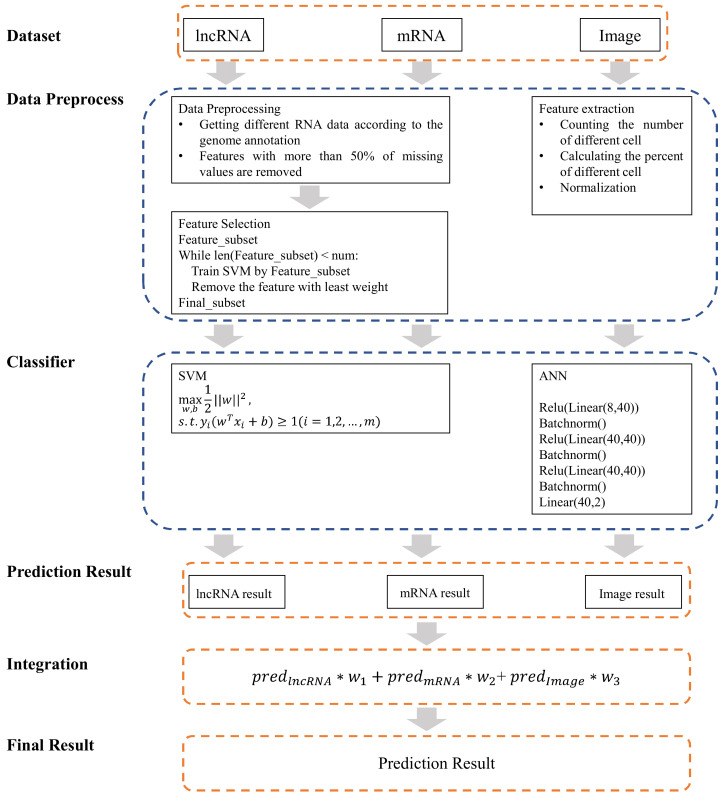
The workflow of the proposed EnsembleSKCM. First, for lncRNAs and mRNAs, data preprocessing was used to remove missing values, and feature selection methods were used to remove redundant features. For images, feature extraction was used to obtain structural features. Next, a support vector machine (SVM) was used to predict metastatic melanoma for lncRNAs and mRNAs, and an artificial neural network (ANN) was used to predict metastatic melanoma for imaging features. Finally, the results from different data modalities were integrated by assigning different weights.

The proposed EnsembleSKCM algorithm integrated the information of lncRNAs, mRNAs and pathology images to detect metastatic melanoma, as shown in Figure 2. A data preprocessing step was used to remove the features, and an additional step of feature selection was used to remove the redundant lncRNA and mRNA features to avoid the overfitting problem [46,47,48]. For image data, a feature extraction step was used to obtain the image feature representation. A three-layer fully connected neural network was designed to detect metastatic melanoma using eight pathology image-based features. The three data sources were then ensembled with different weights to generate the final prediction results.

### 2.4. Feature Selection and Classification Algorithms

Three feature selection algorithms were evaluated in this study, including SVM–RFE [49], variance [50] and t-test [51]. SVM–RFE trained a support vector machine (SVM) model [52,53] and selected features using their weights in the trained SVM model to alleviate the possibility of the “large p small n” paradigm [37]. The redundant features were iteratively removed by the SVM-based recursive feature elimination (SVM–RFE) strategy [49,54,55]. The incremental feature selection (IFS) strategy [56] was used to find the best subset of features ranked by variance (descendent order) or t-test (ascendent order).

This study evaluated six classifiers in the prediction task of metastatic melanoma: random forest (RF) [57], support vector machine (SVM) [58], linear regression (LR) [59], k-nearest neighbor (KNN) [60], decision trees (DT) [61] and naïve Bayes (NB) [62].

### 2.5. Fully Connected Neural Network

A two-layer fully connected neural network was designed to predict metastatic melanoma using pathology image features, as shown in Figure 2. The first layer was designed as $X_{1}=Batchnorm\left(relu\left(linear(X_{0}\right)\right))$, while the second layer was designed as $X_{2}=Batchnorm\left(relu\left(linear(X_{1}\right)\right))$. The third layer was designed as $X_{3}=Batchnorm\left(relu\left(linear(X_{2}\right)\right))$. The output layer was designed as $X_{4}=Batchnorm\left(relu\left(linear(X_{3}\right)\right))$. $X_{0}$ is the input data of the network, and $X_{4}$ is the output data of the network.

### 2.6. Construction of the WGCNA Network

To verify whether image features contribute useful information to the molecular features, the WGCNA analysis was used to calculate the relationship between these two kinds of features. If the correlation between two features was high, these features were redundant to each other. Otherwise, the image features represented useful information for predicting metastatic melanoma. A total of 400 lncRNAs and mRNAs were screened out by the WGCNA package version 1.70–3 [63] in the R-Studio 4.1.3 software.

### 2.7. Implementation Details

The proposed EnsembleSKCM framework integrated multimodal information to predict metastatic melanoma. For lncRNA features, SVM–RFE was used to remove the inter-feature redundancy. The SVM used the linear kernel, and the parameter C was 0.1. SVM–RFE removed one feature per iteration. For mRNA features, SVM–RFE was used to remove redundant features again, and the linear kernel was used. The parameter C was 1. SVM–RFE removed 10 features per iteration due to the very large number of mRNA features. Then, the SVM classifier was used to classify the metastatic version of non-metastatic melanoma. The parameter C was evaluated by the values 0.1, 1, 10 and 100. The four kernels were evaluated, including ‘linear’, ’rbf’, ’poly’ and ’sigmoid’. The grid search was used to screen for the best parameter values. For the image features, we built a two-hidden-layer neural network. Both hidden layers contained 40 neurons. The stochastic gradient descent (SGD) with a batch size of 32 was used to optimize our model for 1000 epochs. The momentum and weight decay parameters were set to 0.9 and 1 × 10^−4^, respectively. The initial learning rate was 0.01. This study was carried out on the Windows 10 operating system with an Intel(R) Core(TM) i7–8750H CPU@2.20GHZ 2.21GHZ and 8 GB RAM.

## 3. Results

### 3.1. Performance of the lncRNA-Based Models

Multiple lncRNAs have been implicated in cancer onset and development [64]. Machine learning methods have been widely used to predict metastatic melanoma [11,12]. In this section, we use the lncRNA biomarkers to predict metastatic melanoma by machine learning methods and investigated which method achieved a better performance. We abbreviated the expression level of an lncRNA as an lncRNA feature in this study.

**Figure 3:**
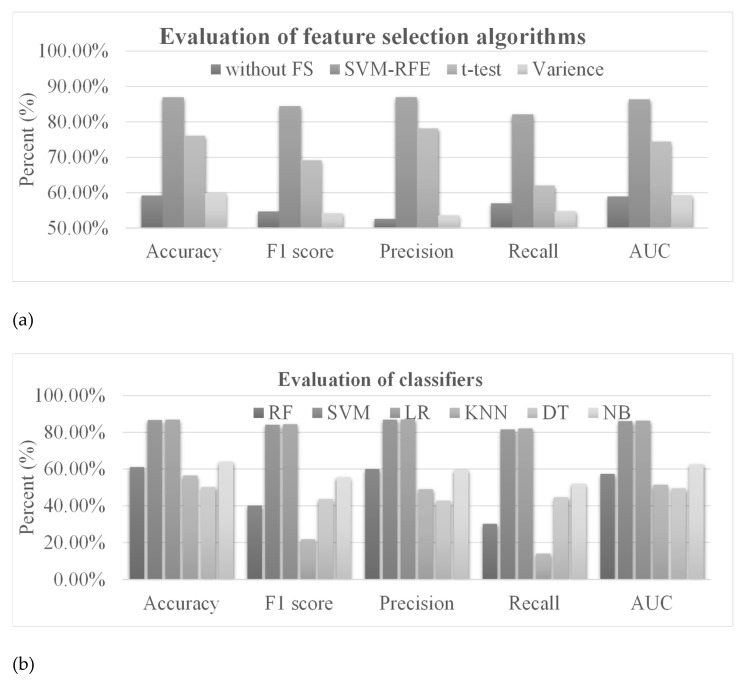

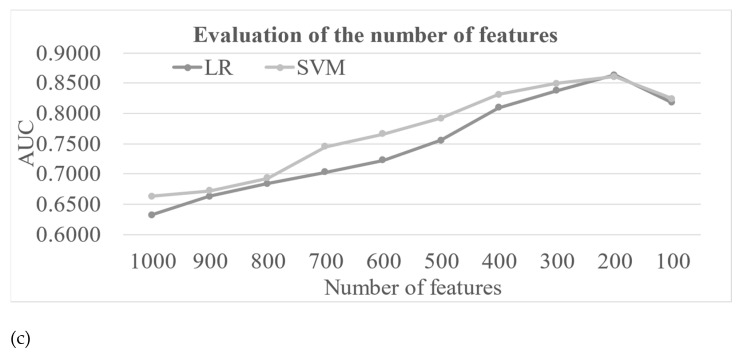
Performance evaluations of the lncRNA-based models. (**a**) Evaluation of the models using three feature selection algorithms and all the features (without FS). The horizontal axis gives the performance measurements. SVM was used as the classifier. Abbreviations: without FS, without the feature selection method; SVM–RFE, support vector machine recursive feature elimination. (**b**) Evaluation of the six classifiers using the SVM–RFE feature selection algorithm. The horizontal axis is the same as in (**a**). Abbreviations: RF, Random Forest; SVM, Support Vector Machine; LR, Linear Regression; KNN, K-Nearest Neighbor; DT, Decision Tree; NB, Naïve Bayes. (**c**) Evaluation of different numbers of features. The horizontal axis gives the number of features used in the SVM–RFE feature selection algorithm. SVM was used as the classifier. Abbreviations: SVM, support vector machine; LR, linear regression.

There were 6919 lncRNAs whose expression levels were profiled in the TCGA–SKCM dataset used in this study, which was much larger than that (414) of the samples. If the expression levels of all these 6919 lncRNAs (also called 6919 lncRNA features) were used to train the prediction models, it would be very easy to overfit the model and a stable prediction performance would not be achieved. We hypothesized that the feature selection methods could remove the redundant features and improve the performance of the prediction model. Therefore, feature selection algorithms were used to screen the lncRNAs whose expression levels were associated with melanoma metastasis [65,66]. Three feature selection algorithms were evaluated using lncRNA features, including SVM–RFE [49], variance [50] and t-test [51]. We used the above feature selection algorithms to select 200 lncRNAs and compared their classification performances with each other. Figure 3a shows that the model using all the features only achieved 0.5918 in accuracy. The prediction accuracy was improved to 0.8696 if SVM–RFE was used to screen the subset of metastasis-related features. SVM–RFE outperformed the t-test and variance in accuracy in selecting features for the prediction task of metastatic melanoma. The best AUC value of 0.8638 was also achieved by SVM–RFE. Therefore, the following section uses SVM–RFE as the feature selection algorithm for the lncRNA data source. Information on all the selected lncRNAs is shown in Supplementary Table S2 in the Supplementary Materials.

The classification algorithm is another important factor for prediction performance. Different models are suitable for different data types. To choose the most suitable model, this study evaluated six classifiers on the prediction task of metastatic melanoma, including random forest (RF) [57], support vector machine (SVM) [58], linear regression (LR) [59], k-nearest neighbor (KNN) [60], decision trees (DT) [61] and naïve Bayes (NB) [62]. Figure 3b shows that SVM and LR achieved the top two best prediction accuracies of 0.8671 and 0.8696, respectively. LR performed slightly better than SVM in both accuracy and AUC. Therefore, the classifier LR was used for the lncRNA data in the following sections.

The number of features selected by the feature selection algorithms was an important factor for prediction performance. The important melanoma-associated features need to be selected, but the redundant features should be eliminated from the final model. SVM and LR achieved similarly good prediction performance, shown in in Figure 3b, and were further evaluated using different numbers of features, shown in in Figure 3c. The AUC values increased with more features eliminated by SVM–RFE until the number of features reached 200. The AUC model decreased to 0.8178 and 0.8243 for LR and SVM, respectively. The best AUC values of 0.8638 and 0.8610 were achieved by LR and SVM using 200 features, respectively. Therefore, 200 was the default number of lncRNAs whose expression levels were chosen for the classification models.

### 3.2. Performance of the mRNA-Based Models

Protein-coding genes represent another important component of the progression of melanoma. This section investigates how the expression levels of mRNAs (also called mRNA features) could facilitate the melanoma metastasis prediction task. 

**Figure 4:**
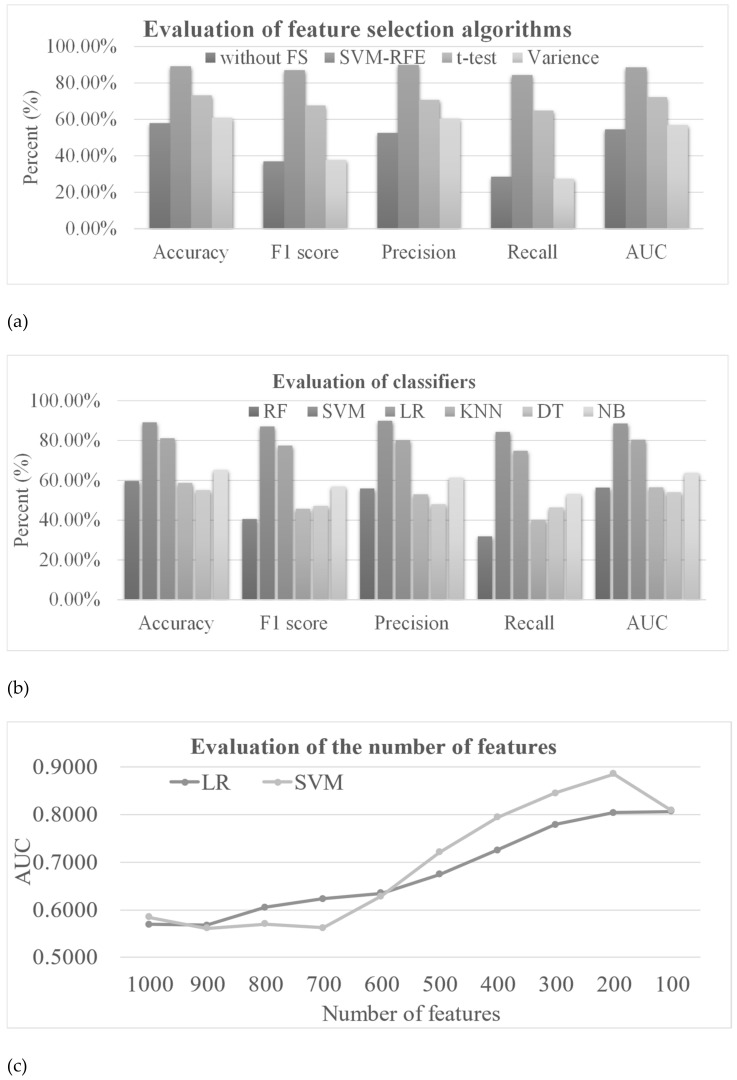
Performance evaluations of the mRNA-based models. (**a**) Evaluation of the models using three feature selection algorithms and all the features (without FS). The horizontal axis gives the performance measurements. SVM was used as the classifier. Abbreviations: without FS, without feature selection method; SVM–RFE, support vector machine recursive feature elimination. (**b**) Evaluation of the six classifiers using the SVM–RFE feature selection algorithm. The horizontal axis is the same as in (**a**). Abbreviations: RF, Random Forest; SVM, Support Vector Machine; LR, Linear Regression; KNN, K-Nearest Neighbor; DT, Decision Tree; NB, Naïve Bayes. (**c**) Evaluation of different numbers of features. The horizontal axis gives the number of features used in the SVM–RFE feature selection algorithm. SVM was used as the classifier. Abbreviations: SVM, support vector machine; LR, linear regression.

There were 19051 mRNAs whose expression levels were profiled in the TCGA–SKCM dataset. This number was also much larger than that (414) of the samples. In order to avoid the overfitting problem, feature selection algorithms were used to reduce the feature dimension. Figure 4a shows that the prediction models using all the mRNA features did not achieve a good performance and the models using the variance-selected features performed only slightly better. The features selected by SVM–RFE achieved the best accuracy of 0.8913, which was 0.3116 better in accuracy than the prediction model using all the mRNA features. Information on all the selected mRNAs is shown in Supplementary Table S3 in the Supplementary Materials.

Different classifiers can achieve different performances in a dataset. This study evaluated six classifiers on how the SVM–RFE-selected features performed on the mRNA-based metastasis prediction task, as shown in Figure 4b. The three classifiers, RF, KNN and DT, only achieved an accuracy smaller than 0.6000, while NB achieved a slightly better accuracy of 0.6522. The other two classifiers, SVM and LR, achieved the best two accuracies of 0.8913 and 0.8116, respectively. The best classifier, SVM, achieved the best AUC of 0.8856.

Different numbers of selected features can influence the prediction performance, as evaluated in Figure 4c. There were fluctuations in the prediction models’ AUC values using 600–1000 features for both SVM and LR classifiers. After the number of features was reduced to less than 600, the prediction AUC values rapidly increased to peaks using 200 features, i.e., an AUC of 0.8041 and 0.8856 for LR and SVM, respectively. The prediction models using 100 features did not achieve the best AUC values. 

### 3.3. Performance of the Image-Based Models

**Figure 5:**
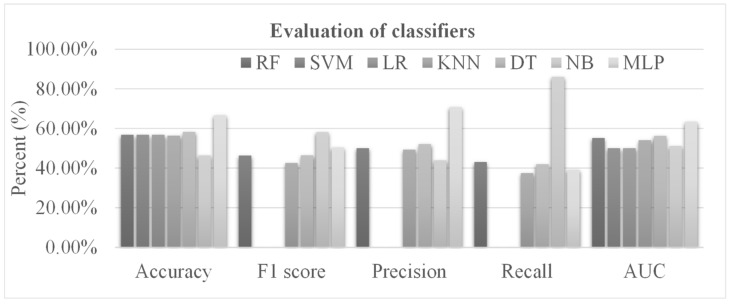
The performance of different classifiers using the features extracted from the pathology images. The horizontal axis lists the performance measurements. The vertical axis gives the values of these measurements. Seven classifiers were evaluated. The performance measurements of some classifiers are zero. Therefore, their corresponding bin heights are 0%, which cannot be displayed in the histogram. Abbreviations: RF, Random Forest; SVM, Support Vector Machine; LR, Linear Regression; KNN, K-Nearest Neighbor; DT, Decision Tree; NB, Naïve Bayes; MLP, Multi-Layer Perceptron.

Pathology imaging provides an important view of cancer tissue and has been widely used in diagnosing primary and metastatic cancers [67,68,69]. Eight cell types were segmented and counted from the pathology images and the percentage of each cell type among all the detected cells was denoted as the image feature of this cell type for the corresponding sample. Figure 5 shows that the six classifiers used for the lncRNA and mRNA features did not achieve accuracies better than 0.6000. Therefore, we further built a fully connected neural network (MLP) for comparison with the six conventional classifiers. The MLP achieved the best accuracy of 0.6667, while the next best classifier, DT, only achieved an accuracy of 0.5821. The MLP’s performance accuracy of 0.6667 was much smaller than those of the lncRNA-based and mRNA-based models. Therefore, we hypothesized that the integration of multi-modal data sources might achieve better metastasis prediction performance.

### 3.4. Integration of Multimodal Data

**Figure 6:**
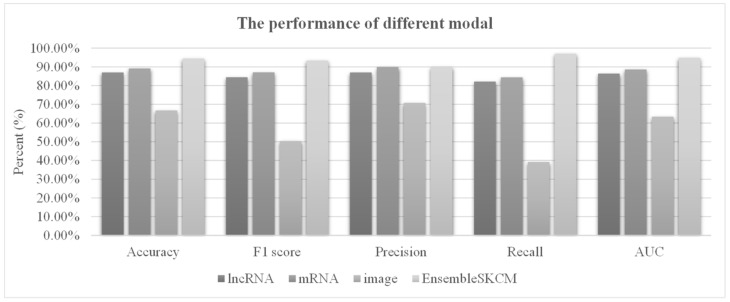
Contribution comparison of multimodal data for the metastasis prediction task. The horizontal axis lists the performance measurements. The vertical axis gives the values of the measurements. Three modalities were evaluated, including lncRNA, mRNA and image. lncRNA, mRNA and image features were used to train models and showed the best performance. Their integration was denoted as the proposed EnsembleSKCM model. The best model of each data modality was used. For each measurement, integrating all the data modalities can achieve the best performance.

All three data modalities (lncRNA, mRNA and image features) contributed useful information for melanoma metastasis, and we hypothesized that their integration may obtain better prediction performance. Figure 6 supports the necessity of integrating multimodal data for the metastasis prediction task. Both lncRNA and mRNA features facilitated the prediction models with accuracies >0.8500, while the prediction model using the image-extracted features only achieved an accuracy of 0.6667. After the integration of all three data modalities, EnsembleSKCM achieved a much better prediction accuracy of 0.9444, improving the three lncRNA, mRNA and image data modalities by 0.0749, 0.0531 and 0.2778 in accuracy, respectively.

### 3.5. Integration of Image Features with Molecular-Level Features

Molecular-level data fully reflected the genetic information of melanoma, while image features represented the macrolevel information. To verify our ensembling hypothesis, we integrated the image features with the molecular-level features and evaluated the integration performances. As shown in Table 1, the image features may improve the model based on mRNA features by 0.0024 in accuracy and 0.0211 in AUC. The model based on lncRNA features may be improved via the integration of the image features by 0.0023 in AUC, with a slight decrease of 0.0049 in accuracy. The improved parameter-independent AUC suggests that integrating the image features provides a more balanced prediction performance. The proposed EnsembleSKCM model integrated all three data sources and improved the model using only the mRNA and lncRNA features by 0.0024 in accuracy and 0.0030 in AUC. The above data suggest the importance of adding pathological imaging features for predicting metastatic melanoma.

### 3.6. Correct Prediction of Samples Using Different Data Modalities

We further investigated the details of how different modalities facilitated the metastasis prediction task, as shown in Table 2. All three modalities led to metastasis prediction models with satisfying numbers of correctly predicted positive samples, i.e., primary melanoma. The lncRNA-based and mRNA-based models correctly detected approximately 0.8100 metastatic melanomas, while the image-based model correctly detected only 0.3911 metastatic melanomas. However, the image-based features represented an important view of metastatic melanoma, and its integration with the lncRNA and mRNA features improved the ensembled model to 0.8994 in the percentage of correctly predicted metastatic melanoma samples.

### 3.7. Comparison of EnsembleSKCM with Existing Metastatic Melanoma Prediction Methods

Metastatic melanoma is a high-risk cancer, and several machine learning methods have been published to predict metastatic melanoma. Bellomo et al. used the logistic regression algorithm to combine the clinicopathologic and gene expression features to predict sentinel lymph node metastatic melanoma [10]. They achieved a prediction AUC of 0.82. Garg et al. used random forest trained with signature genes to predict metastasis and achieved the best AUC of 0.68 [11]. Mancuso et al. classified early-stage melanoma patients with high and low risk of metastasis and achieved an AUC of 0.8922 [12]. Shepelin et al. used SVM to identify 44 characteristic signaling pathways associated with melanoma metastasis [13]. Their model achieved accuracies of 0.94 for metabolic pathways and 0.923 for signaling pathways. As summarized in Table 3, the proposed EnsembleSKCM model outperformed the existing methods based on the AUC and accuracy performance metrics.

### 3.8. Analysis of the Relationship between Molecular and Image Features Using WGCNA

**Figure 7:**
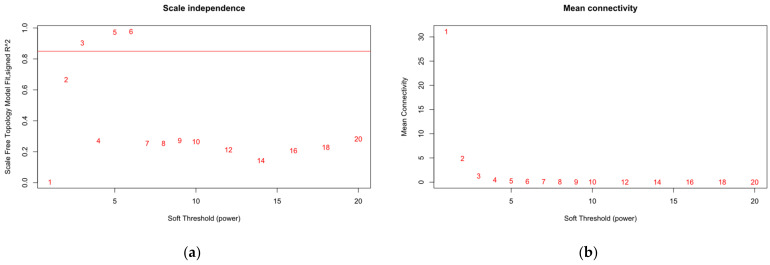

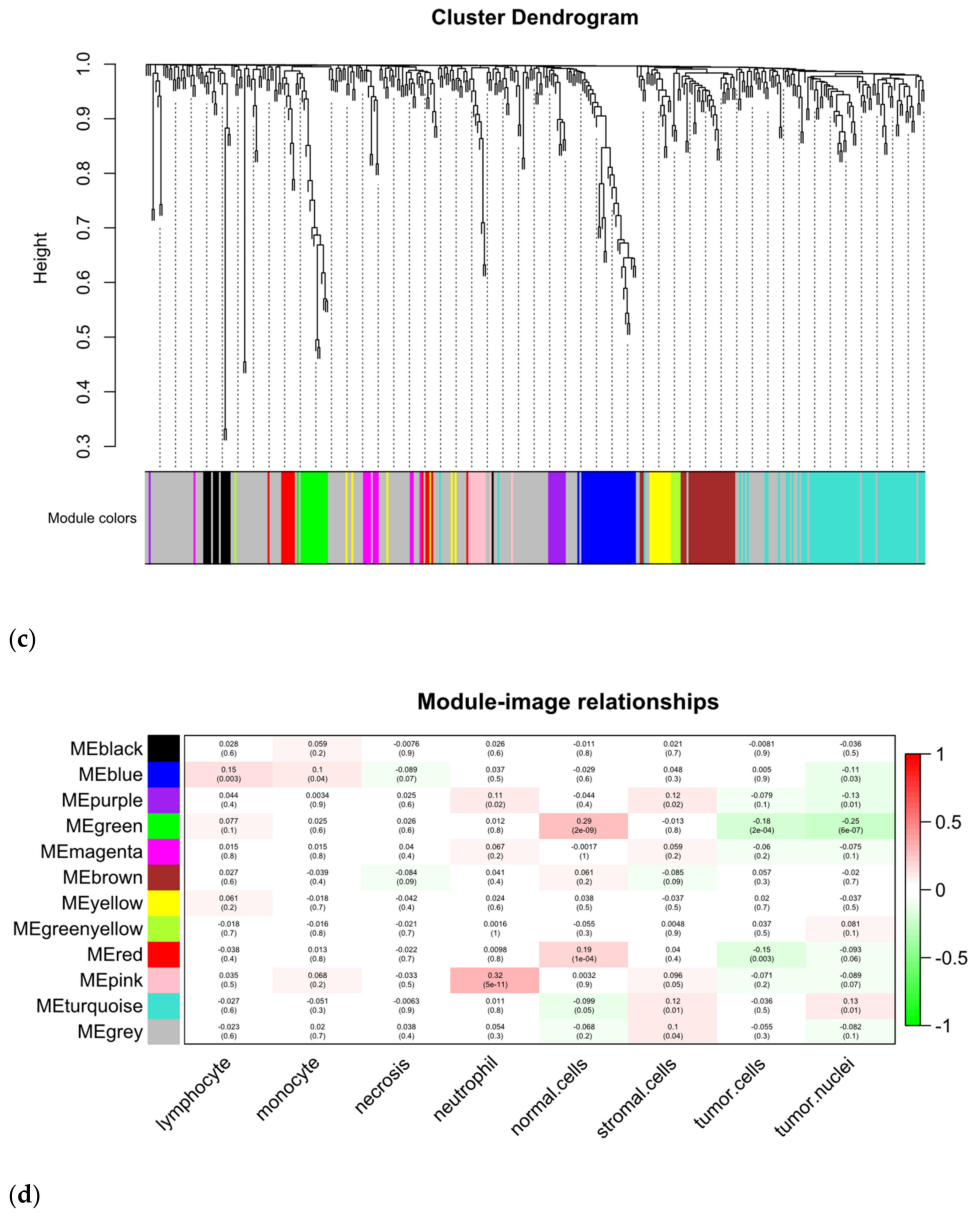
WGCNA analysis. (**a**) The network topology for different soft-threshold powers. This image shows the scale-free topology fit index influenced by soft-threshold power. The scale-free topology index was 0.85, which conformed to the power law distribution. (**b**) The network topology for different soft-threshold powers. This image shows mean connectivity influenced by soft-threshold power. (**c**) Gene clustering tree using hierarchical clustering of adjacency-based dissimilarity. (**d**) The module–image relationships. The correlation between the imaging features and molecular features was low; imaging features can provide complementary information to the prediction of metastatic melanoma.

To verify whether image features were redundant to molecular features, the WGCNA analysis was used to calculate the relationship between two kinds of features. As shown in Figure 7a, the soft-threshold power was defined as 3 and the scale-free topology index was 0.85, which conformed to the power law distribution. As shown in Figure 7b, when the soft threshold is 3, the curve tends to smooth and proves the good network connectivity. The gene dendrograms and respective module colors are shown in Figure 7c. We divided the molecular features into 12 modules. Figure 7d shows the relationship between the molecular modules and image features. The strongest correlation coefficient of 0.32 (*p* = 5 × 10^−11^) was observed between neutrophils and MEpink. We also provide information about the genes corresponding to each module in the Supplementary Table S4. There were only a few other significant correlations between the molecular modules and the image features. Therefore, most of the imaging features could contribute nonredundant complementary information to the prediction of metastatic melanoma.

### 3.9. Validation of the Results in Another Cohort

To further verify the validity of the model, another cohort was used to test the model. The classification models were trained using TCGA samples and tested using the GSE59455 dataset. Table 4 evaluates different classifiers using the two data sources, lncRNA and mRNA, and their integration. The classifier GBDT achieved the best accuracy of 0.6333 using the combined list of lncRNAs and mRNAs, which improved the two GBDT models using lncRNAs and mRNAs, separately. The prediction accuracies had large room for improvement due to the variations between the two transcriptomic profiling technologies’ array and RNA-seq. However, the overall data support the observation that lncRNAs and mRNAs contribute complementary information to each other, and their combination leads to better prediction models.

The same stratified five-fold cross-validation (S5FCV) strategy was used to evaluate the proposed EnsembleSKCM algorithm on the new GSE59455dataset, as shown in Table 5. The top 10 lncRNAs and top 10 mRNAs ranked by t-test were evaluated. The classifier NB achieved the best models on both lncRNA (Acc = 0.9500) and mRNA (accuracy = 0.8333) features, while the best prediction model (Acc = 0.9667) was achieved by combining the lncRNA and mRNA features. In summary, NB and all the other classifiers supported the importance of combining the complementary data sources of lncRNAs and mRNAs.

## 4. Discussion

This study proposed the EnsembleSKCM framework to integrate the data modalities of lncRNA, mRNA and pathology images for the prediction of metastatic melanoma. The data suggest that each data modality represents an important view of the metastatic melanoma.

Some lncRNAs are known to be closely associated with the prognosis of melanoma [30,64]. Machine learning methods have already been utilized to investigate how lncRNAs are involved in the prognosis of melanoma [33]. Not all lncRNAs contributed to metastatic melanoma and the experimental design supported this through feature selection algorithms. 

The view of lncRNA features alone did not achieve a satisfying prediction performance of metastatic melanoma. Therefore, the mRNA features and image-based features were also evaluated for single-modal prediction performance. The mRNA-based model achieved a similar performance as the lncRNA-based model, while the image-based model achieved a much worse performance. 

However, the integration of all three data modalities generated the best model, with an accuracy rate of 0.9444. The experimental data suggest that the multimodal EnsembleSKCM model outperformed the models using only single-modal data, although the image-based model only achieved 0.3911 in the percentage of correctly predicted metastatic melanoma samples.

The lncRNA–mRNA interaction network described the close connections between the two data modalities, lncRNA and mRNA, and novel insights could be derived from the network view about melanoma compared with studies using only one modality [31,70]. This study further integrated the macrolevel image features with the molecular-level lncRNA and mRNA features. The experimental data suggest that the integration of these three data modalities may further improve the prediction performance of metastatic melanoma.

WGCNA was used to analyze the relationship between the molecular features and the image features. The data suggest that there are limited correlations between molecular and image features. Therefore, it is important to integrate both molecular and imaging features for a better prediction of metastatic melanoma.

We compared our method with the existing metastatic melanoma prediction methods. The comparison data suggest the necessity of integrating lncRNA, mRNA and image features for the prediction of metastatic melanoma. The integrated model of the three data modalities also outperformed the existing studies in this task.

Due to the limitation in data availability, the dataset used in this study is already the largest cohort. The ideal validation cohort consists of melanoma patients with paired samples before and after metastasis, and the transcriptomes are profiled by RNA-seq technology. A minimum requirement is a cohort of gender- and race-matched patients with metastatic and non-metastatic melanoma, considering the gender- [71,72] and racial disparities [73]. We only found an array-based transcriptomic dataset of metastatic melanoma to validate our method. The experimental results of both the TCGA-trained model and the proposed EnsembleSKCM algorithm support the importance of combining the complementary lncRNA and mRNA data sources. In addition to the lncRNA, mRNA and pathology image features, dermoscopic images and somatic mutations may also be considered in the integrated EnsembleSKCM framework in future studies. 

This study quantitatively suggested that the precise diagnosis of metastatic melanoma may need to integrate complementary information from both molecular and macroscopic features, including lncRNAs, mRNAs and pathology images. These features represent the dynamic situations of melanoma lesions. In future research, in addition to integrating dynamic lncRNA and mRNA features, other slowly altered features, such as somatic mutations, will be evaluated in the integrated EnsembleSKCM framework for their contributions to the performance of metastatic melanoma prediction. In addition, more handcrafted feature types will be considered for the pathology images. Deep neural networks are good at automatically learning the latent patterns within images and will also be utilized to extract useful features from pathology images for the metastatic melanoma prediction task.

Additional RNA-seq transcriptomic datasets together with pathological images unbiased across multiple ethnic groups will be sought to further validate our proposed model in future studies.

## 5. Conclusions

This study extensively evaluated metastatic melanoma prediction models using three data modalities. The experimental data support the necessity of removing redundant features and testing different classifiers. The integration of all three data modalities also improved the single-modal models by at least 0.0531 in prediction accuracy. Metastatic melanoma has a high mortality rate, and the recently developed immunotherapy has produced major clinical success in treating lethal melanoma [74]. The precise risk assessment of melanoma provides important information for deciding follow-up treatment plans, including immunotherapy. Therefore, it is both clinically important and computationally challenging to develop precise risk assessment models for melanoma [75,76].

There are still limitations remaining in the proposed model. We trained and validated our model across different transcriptome profiling platforms (RNA-seq and microarray). Although the cross-platform validation results show that our detected transcriptome biomarkers delivered satisfactory melanoma metastasis prediction performances, the integrated model of the three data sources (lncRNA, mRNA and image) remains to be evaluated on an independent cohort. Melanoma has a 12-times higher incidence rate in the United States than in China [77]. Therefore, an independent cohort across different ethnic group will be recruited to cover the multi-modal data sources in the future studies. The experimental evaluation of the validity and robustness of our EnsembleSKCM model is also worth future studies for the detection of metastatic melanoma in the clinical practice.

System biology models have the capability of integrating heterogeneous data sources in network settings. We plan to explore the possibility of combining systems biology and machine learning approaches via the graph convolutional network for the prediction of melanoma metastasis.

## Figures and Tables

**Table 1:** How the image features influence the performance of the model. mRNA + Image indicates the performance of integrating mRNA and image features. mRNA indicates the performance of mRNA features. LncRNA + Image indicates the performance of integrating lncRNA and image features. LncRNA indicates the performance of LncRNA features. LncRNA + mRNA indicates the performance of integrating lncRNA and mRNA features. EnsembleSKCM indicates the performance of all features.

	mRNA + Image	mRNA	LncRNA + Image	LncRNA	LncRNA + mRNA	EnsembleSKCM
Accuracy	0.8937	0.8913	0.8647	0.8696	0.9420	0.9444
F1 score	0.8659	0.8703	0.8372	0.8448	0.9306	0.9333
Precision	0.7933	0.8988	0.8045	0.8698	0.8994	0.8994
Recall	0.9530	0.8436	0.8727	0.8212	0.9641	0.9699
AUC	0.9067	0.8856	0.8661	0.8638	0.9456	0.9486

**Table 2:** The numbers of correctly predicted positive and negative samples using different modalities. The “Total” column gives the total numbers of positive and negative samples. The “lncRNA”, “mRNA” and “image” columns give the data for the individual data modalities. The last column gives the numbers of correctly predicted positive (true positives, TP) and negative (true negative, TN) samples using the multimodal EnsembleSKCM model.

	Total	lncRNA	mRNA	Image	EnsembleSKCM
TP	235	214	225	206	230
TN	179	146	145	70	161

**Table 3:** Comparison of EnsembleSKCM with other studies. The “Publication” column gives the authors’ names and the publication date. The “Methods” column gives the method used in this study. The “Key Finding(s)” column gives the main findings of this study. The “Performance” column gives the value of evaluating indicator.

Publication	Methods	Key Finding(s)	Performance
Bellomo et al., 2020 [10]	Logistic regression model optimized by penalized maximum likelihood estimation algorithm	The model combining clinicopathologic and gene expression features better predicted SLN metastases than only one type of above features	AUC = 0.82
Garg et al., 2021 [11]	Random Forest	The machine learning models trained with signature genes performed better in predicting metastases than models trained with clinical covariates or published prognostic signatures	AUC = 0.68
Mancuso et al., 2021 [12]	Logistic Regression, Support Vector Machine, Decision Tree, Gaussian Naïve Bayes, K-Nearest Neighbors	The machine learning method that classified early-stage melanoma patients with high and low risk of metastasis by serum cytokines and Breslow thickness can best predict metastatic melanoma	AUC = 0.8922. Accuracy = 0.8502
Shepelin et al., 2018 [13]	SVM	Identified 44 characteristic signaling pathways associated with metastatic melanoma	Accuracy (metabolic pathways) = 0.94 Accuracy (signaling pathways) = 0.923
Ours	EnsembleSKCM	Integrates LncRNA, mRNA and image features to obtain better performance in recognizing metastatic melanoma	Accuracy = 0.9444. AUC = 0.9486.

**Table 4:** Accuracies of different modalities by different classifiers. The “mRNA” row gives the prediction accuracies of the mRNA-based classification models. The “lncRNA” row gives the prediction accuracies by different classifiers based on lncRNA data. The “EnsembleSKCM” row evaluates different classifiers by combining both data sources. The prediction model was trained using TCGA samples and tested on array-based transcriptomic samples from GSE59455.

	NB	GBDT	SVM	KNN	DT	LR	RF
mRNA	0.3000	0.5333	0.4667	0.4500	0.5167	0.4500	0.3667
lncRNA	0.3000	0.5333	0.2833	0.4500	0.4167	0.2833	0.4167
EnsembleSKCM	0.3000	0.6333	0.4667	0.5000	0.5167	0.4500	0.4333

**Table 5:** Accuracies of different modalities by different classifiers. The “mRNA” row gives the prediction accuracies of the mRNA-based classification models. The “lncRNA” row gives the prediction accuracies by different classifiers based on lncRNA data. The “EnsembleSKCM” row evaluates different classifiers by combining both data sources. The prediction accuracies were calculated using the stratified five-fold cross-validation (S5FCV) strategy.

	NB	GBDT	SVM	KNN	DT	LR	RF
mRNA	0.8333	0.7167	0.7167	0.8167	0.6167	0.7167	0.8000
lncRNA	0.9500	0.8500	0.9333	0.8000	0.8500	0.9333	0.9000
EnsembleSKCM	0.9667	0.8500	0.9333	0.8500	0.8500	0.9500	0.9167

## Supplementary Materials

The following supporting information can be downloaded at: https://www.mdpi.com/article/10.3390/genes13101916/s1, Table S1. The metadata information of the TCGA dataset. The sample names were in the column “Sample”. The classes of “Primary” or “Metastasis” were in the column “Class”. The other metadata are listed in the other columns. Table S2. Summary of LncRNA selected by the feature selection algorithm. The ensembl IDs were given in the column “ENSEMBL”, the chromosome names were given in the column “Chr”, the start coordinates of genes were given in the column “Start”, the end coordinates of genes were given in the column “End”, the gene names were given in the column “SYMBOL”. Table S3. Summary of mRNA selected by the feature selection algorithm. The ensembl IDs were given in the column “ENSEMBL”, the chromosome names were given in the column “Chromosome”, the start coordinates of genes were given in the column “Start”, the end coordinates of genes were given in the column “End”, the gene names were given in the column “SYMBOL”. Table S4. The information about the gene corresponding to each WGCNA module. The ensembl IDs were given in the column “Gene Name”, the module names were given in the column “WGCNA-Module”.
